# Influence of Structural Properties of Oleic Acid-Capped CdSe/ZnS Quantum Dots in the Detection of Hg^2+^ Ions

**DOI:** 10.1007/s10895-024-03828-0

**Published:** 2024-07-16

**Authors:** Fredy Giovany Ortiz Calderon, Brayan Stiven Gómez Pineros, Nathan D. McClenaghan, Gilma Granados-Oliveros

**Affiliations:** 1 Grupo de Síntesis Orgánica Sostenible, Departamento de Química, Facultad de Ciencias, Universidad Nacional de Colombia, Bogotá, DC Colombia; 2 Institut des Sciences Moléculaires, CNRS UMR 5255, University of Bordeaux, Talence, 33405 France

**Keywords:** CdSe/ZnS quantum dots, Detection of Hg^2+^ ions, Quenching and enhancing of fluorescence

## Abstract

Oleic acid-capped CdSe/ZnS quantum dots (QDs) were used to investigate their photoluminescence (PL) response to Hg^2+^ ions as a function of the surface properties of QDs. Three distinctly-size CdSe/ZnS QDs were obtained by varying the molar ratio of shell precursors, which were characterized by X-ray diffraction (XRD), high-resolution transmission electron microscopy (HR-TEM), Fourier-Transform infrared (FT-IR), X-ray photoelectron spectroscopy (XPS), absorption spectroscopy, and time-resolved fluorescence spectroscopy. Results revealed the obtention of zinc blende nanocrystals with sizes ranging from 2.7 to 3.2 nm (± 0.5) and ZnS thickness between 0.3 and 1.0 monolayer (ML). The variation of the [S]/[Zn] molar ratio introduced chemical species that act as traps, affecting the PL properties differently. Depending on the thickness of the shell and chemical speciation on surface, Hg^2+^ ions could induce quenching or enhancement of PL. Detection of mercury ions was evaluated in terms of Stern-Volmer equation, where the limit of detection (LOD) for the PL quenching system was 11.2 nM, while for the PL enhancing systems were 8.98 nM and 10.7 nM. Results demonstrate the performance of oleic acid-capped CdSe/ZnS QDs to detect Hg^2+^ and their capacity to turn the PL on/off depending on surface properties.

**Supplementary Information:**

The online version contains supplementary material available at 10.1007/s10895-024-03828-0.

## Introduction

Quantum dots (QDs) of semiconductors are nanocrystals with particle sizes between 1 and 10 nm, which have found applications in different fields, such as sensors, lasers, photovoltaics, and light-emitting diodes [1]. II-VI semiconductors like CdSe are mainly characterized by a broad absorption spectrum and a narrow and intense emission that could be multicolor since it depends on their size due to the quantum confinement effect [2]. These characteristics are very appreciated for metal ion sensing applications [3, 4].

Indeed, for QD-based metal ion detection, CdSe QDs have been modified with different ligands to avoid aggregation, enhance stability, and improve their selectivity and sensitivity [5]. With this goal, the CdSe QDs surface has been modified with hydrophilic ligands, resulting in a fluorescence signal intensity that could be quenched or enhanced as a response to metal ions [5, 6]. For instance, quenching of fluorescence has been reported with L-cysteine-capped CdSe QDs to detect Pb^2+^ions [7], mercapto acetic acid-capped CdSe QDs have been applied in the detection of Ag^+^ ions [8], and mercaptoethanesulfonate-capped CdSe QDs for Cu^2+^ determination [9]. Equally, enhancement of PL has been reported with L-cysteine-capped CdSe to detect Zn^2+^ ions [9] and L-carnitine-capped CdSe/ZnS QDs to detect Hg^2+^ [10]. Other QD types have also been reported considering the enhancement of PL intensity induced by cations. For example, mercaptopropionic acid-capped CdTe QDs in the presence of Cd^2+^ [11], mercaptoethanol-capped PbS QDs in the presence of mercapto acetic acid-capped InP QDs in the presence of Hg^2+^ ions [12] and L-cysteine capped CdS QDs in the presence of Ag^+^ ions [13].

Mercury ions, the most potent neurotoxin in human physiology, are selected as analyte in this investigation due to the severe environmental and public health problems in counties that use mercury in gold extraction mining. Many fluorescent-based optical sensors [14] have been applied to detect Hg^2+^ ions in real time with high sensitivity, selectivity, and signal-to-noise ratio [15].

For QDs-based mercury ion sensors, the interaction between analyte and nanocrystals leads to many pathways that affect photoluminescence intensity. According to the affinity of metal ions and QDs, cation exchange could be produced by replacing cations of starting QDs with cations of the analyte, preserving the original anion sublattice [16]. This cation exchange reaction forms small particles on the QDs surface, causing non-radiative recombination of excited electrons and holes that finally quenches fluorescence.

Conversely, the strategies reported by literature that led to increased fluorescence for determining cation ions are less frequent. Surface state passivation of QDs removes the traps that facilitate non-radiative recombination pathways [17]. Understanding the role of trap states is challenging. In its simplest form, a trap can be described as a stable nonbonding orbital of an undercoordinated atom. This orbital usually lies deep in the bandgap, where it can act as an electron or hole trap. Metal ions on QDs surface can produce non-coordinative atoms and dimers [18], indirectly affecting the number of dangling bonds and the presence of traps [19].

In this context, the growth of a shell of semiconducting material with a larger bandgap (3.7 > 1.7 eV) [2] than CdSe, such as ZnS, plays a determinant role since the shell passivates the traps and defects of core [20, 21], improving stability [1, 20], and photoluminescence [2, 22, 23]. This type of QDs is known as a type I core/shell system, characterized by the confinement of the exciton (an electron-hole pair) to the core [24]. Type I structures are the most studied in analytical applications since this configuration provides the best confinement of the exciton (an electron-hole pair) and the highest rates of radiative electron-hole recombination (i.e., the brightness of the photoluminescence) [25]. However, excess shell (greater than 2.2 monolayers [26]) can cause the opposite effect in the PL of core/shell QDs, because the lattice mismatch between CdSe and ZnS is 12%, which generates a strain at interface [27] that creates non-radiative recombination sites, impairing the properties of PL [21, 28, 29].

Our group has previously studied Hg^2+^ detection by PL measurements using CdSe/ZnS QDs modified with two ligands of differing water affinity, such as L-glutathione and oleic acid [30]. Both QDs produced PL quenching. Wang et at. [31] reported the fluorescence enhancement induced by Hg^2+^ using CdSe/ZnS QDs. The enhanced PL was attributed to Zn^2+^/Hg^2+^ cation exchange in the ZnS shell, producing Hg_x_Zn_1−x_S/CdSe, increasing the separation of electrons and holes, and reducing the recombination rate.

Since oleic acid-capped CdSe/ZnS could detect metal ions, we used these nanomaterials to induce PL quenching and enhancement. For this, the present investigation used the variation of the molar ratio of shell precursors to obtain three nanocrystals with different shell thicknesses, forming traps that affected PL properties in different ways. We discuss the fluorescence response of QDs regarding the shell thickness, [S]/[Zn] molar relation, surface properties, and PL decay dynamics. Surface properties are characterized by FT-IR, XPS, TEM, and HR-TEM measurements. Results show that an adequate molar ratio of shell precursor could passivate the trap sites of surface QDs. In addition, a thicker ZnS shell (0.7-1.0 ML ranging) protects the core from exchange cation reactions, avoiding the quenching of PL in the presence of Hg^2+^ ions. Thus, we demonstrate the shell dependence to turn PL on/off induced by mercury ions and other metal cations.

## Experimental Section

### Materials

Cadmium oxide powder (99.9%), trioctylphosphine (TOP, 99%), selenium powder (99.9%), hexamethyldisilathiane ((TMS)_2_S, 99%), diethylzinc (Et_2_Zn, 1.0 M/hexane), oleic acid (OA), and 1-octadecene (ODE, tech. 99%) were purchased from Sigma-Aldrich. Metal chloride (HgCl_2_, MnCl_2_, CoCl_2_, NiCl_2_, ZnCl_2_, CdCl_2_, PbCl_2_), ethanol (99.5%), acetone (99.8%), methanol (99.5%), and acetonitrile (CH_3_CN, 99%) were obtained from Panreac. All reagents were analytical grade and used without further purification. Ultrapure water (18 MΩ) was used to prepare aqueous solutions.

### Synthesis of Oleic Acid-Capped CdSe/ZnS QDs

The CdSe/ZnS QDs were prepared using a method previously [30], and the details of the synthesis process are provided in the supporting information. Three CdSe/ZnS samples with varied sizes were obtained by changing the amount of Et_2_Zn to 31 µL (0.031 mmol), 140 µL (0.14 mmol), and 280 µL (0.28 mmol), while the amount of (TMS)_2_S was kept constant (0.28 mmol).

### Characterization

#### HR-TEM Analysis

The High-Resolution Transmission Electron Microscopy (HR-TEM) images of the QDs were taken using a Tecnai F20 Super Twin TMP Microscope, Field emission source, resolution of 0.1 nm at 200 kV, maximum magnification at TEM 1.0 MX, GATAN US 1000XP-P camera. EDX Oxford Instruments XMAX detector. STEM Analysis - FISCHIONE Instruments Model M3000 FP5360/22 HAADF Detector 120/200 kV. The samples were dispersed in chloroform using an ultrasonic bath. After, a drop was deposited on a carbon film grid; then, the sample was dried and analyzed in the transmission microscope using 450.000 x magnification. Image-J software was used to calculate the average diameter and d-spacing [111] of QDs with HR-TEM micrographs.

The shell thickness parameter is the difference between the size of QDs core-shell (CdSe/ZnS) and size core (CdSe) from HRTEM analysis (Eq. 1).

1 $$\eqalign{ Shell\,thickness\, = \, & Size\,CdSe\,/\,ZnS\,QDs \cr & - \,Size\,CdSe\,core\,QDs \cr}$$

The monolayer number (ML) was calculated as the ratio value between the experimental shell thickness parameter and ZnS zinc blende theoric monolayer size (0.70 nm) (Eq. 2) [32].

2 $$\eqalign{& Monolayer\;number\;\left( {ML} \right) \cr & = {{shell\;thickness\;experimental} \over {0.70\;nm}} \cr}$$

#### XRD Patterns

Panalytical X’Pert3 Pro Multipurpose Diffractometer with CuK_α_ radiation source (45 kV and 40 mA) was employed to measure the powder XRD patterns. The diffraction dataset cards from the Joint Committee of Powders Diffraction Standards (JCPDS) were used to compare the obtained patterns.

#### FT-IR Analysis

Infrared spectra of quantum dots were recorded with a Thermo Nicolet Nexus 670 FTIR spectrometer at a resolution of 4 cm^–1^ using a KBr pellet, summing 50 scans.

#### X-ray Photoelectron Spectroscopy (XPS)

The XPS experiments were recorded using the XPS/ISS/UPS- ACenteno surface characterization platform built by SPECS (Germany). The platform is equipped with a PHOIBOS 150 2D-DLD energy analyzer. A monochromatized Al Kα X-ray source (FOCUS 500) operated at 100 W was used for the measurements. The pass energy of the hemispherical analyzer was set at 100 eV for general spectra and 20 eV for high-resolution spectra. The samples were mounted on copper conductive tape in stainless steel metal sample holders for analysis and provided by the manufacturer SPECS. These sample holders are electrically connected to the spectrometer. CasaXPS program (Casa Software Ltd) for data analysis and the SPECS Prodigy library for RSF values were used.

#### UV-Vis Spectroscopy

Electronic absorption spectra were measured on a UV-Vis spectrophotometer EMC-11-UV. Samples were measured in dilute solution in 1 cm pathlength quartz cells.

#### Photoluminescence (PL) Measurements

Steady-state PL measurements were performed in an Agilent Varian Cary Eclipse Fluorescence spectrophotometer, using optically dilute solutions in 1 cm x 1 cm quartz cells. The PL quantum yield (PLQY, Φ) of CdSe/ZnS QDs (dissolved in chloroform) was calculated using rhodamine 6G in methanol solution as standard (Φ_s_ = 94%), with excitation wavelength (*λ*_*exc*_) at 500 nm. Equation 3 was employed to calculate PLQY [33].

3 $${\Phi _x}\, = \,{\Phi _s}\,\left( {{{O{D_s}} \over {O{D_x}}}} \right)\left( {{{{I_x}} \over {{I_s}}}} \right)\left( {{{n_x^2} \over {n_s^2}}} \right)$$

OD is the optical density (absorption) at the excitation wavelength, $I$ is the area under the emission band, $n$ is the refractive index of the solvent, s is the standard solution, and $x$ are values of the unknown solution.

#### Time-Resolved Photoluminescence (TRPL)

TRPL measurements were carried out using a Horiba Fluorolog Time-Correlated Single Photon Counter (TCSPC) system, using a 560 nm nano LED as the pulsed excitation source (1 MHz; FWHM = ca. 1 ns). Measurements were performed at room temperature.

### Changes Optical Induced by Hg^2+^

Hg^2+^ detection was carried out as follows: 100 µL of Hg^2+^ ions in an aqueous solution (0.3 mM − 5.0 mM) were added to 3000 µL of a solution containing CdSe/ZnS QDs (240 ppm) dissolved in chloroform and ethanol (1:1). The mixtures containing Hg^2+^ and QDs systems were stirred at room temperature, and then PL spectra were measured using *λ*_*exc*_ = 380 nm, slit: 3 (excitation) and 2 (emission), integration time: 0.3 s.

#### Effect of Other Metal Ions

The changes in the fluorescence intensity originated from several metal ions (Mn^2+^, Co^2+^, Pb^2+^, Ni^2+^, Ba^2+^, Cd^2+^, and Zn^2+^) and were determined with experimental conditions similar to the detection of Hg^2+^. In a typical experiment, 100 µL of metal salt in an aqueous solution (5.0 µM) were added to 3000 µL of CdSe/ZnS QDs dissolved in a CHCl_3_/EtOH 1:1 (240 ppm). The mixtures containing cations and QDs systems were stirred at room temperature, and then PL spectra were measured using *λ*_*exc*_ = 380 nm, slit: 3 (excitation) and 2 (emission), integration time: 0.3 s.

## Results and Discussion

### Structural Characterization

#### TEM and HR-TEM Analysis

**Fig. 1:**
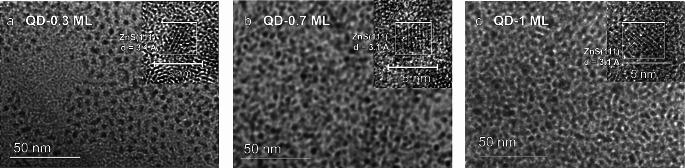
TEM and HR-TEM (inset) micrographs of CdSe/ZnS QDs samples: a) QD-0.3 ML, b) QD-0.7 ML, c) QD-1.0 ML

CdSe QDs were synthesized by injecting Cd and Se precursors into a hot 1-octadecene and oleic acid solution. The growth of the ZnS shell around CdSe core was performed by a reaction of Et_2_Zn and (TMS)_2_S at 80 °C. The molar ratio of [S]/[Zn] precursors was 9.0, 2.0, and 1.0 to obtain particles with variations in the size and thickness of the shells (Fig. 1; Table 1). From TEM and HR-TEM images, the average diameters of QDs are 2.7 nm, 3.0 nm, and 3.2 nm (± 0.5 nm), corresponding to an amount of ZnS monolayer (ML) of 0.3 (QD-0.3 ML), 0.7 (QD-0.7 ML), and 1.0 nm (QD-1 ML), respectively. Figure S2 shows the size distribution obtained from TEM micrographs. ZnS monolayer around CdSe was calculated from the difference between the core diameter (2.5 nm) from HR-TEM images shown in Figure S1 and the theoretical value of one monolayer (Eqs. 1 and 2) [32]. TEM images also revealed the obtention of spherical structures with good crystallinity and reasonable, narrow size distributions. There is no evidence of an interface between the core and shell, meaning that the shell growth process occurs in a coherent epitaxial region, as described in previous reports [21].

**Table 1:** Size and MLs of core and core/shell QDs

QDs	[S]/[Zn] molar ratio	Average diameter [nm]	ZnS shell thickness [nm]	ZnS MLs
CdSe	0	2.5 nm	0	0
QD-0.3 ML	9/1	2.7 ± 0.5	0.2	0.3
QD-0.7ML	2/1	3.0 ± 0.6	0.5	0.7
QD-1ML	1/1	3.2 ± 0.5	0.7	1.0

Crystal phases of QDs were determined with the interplanar distance spacing of selected areas in the HRTEM images, applying the Fast Fourier Transform (FFT) of ImageJ software (in Fig. 1, micrograph insets). The interplanar spacing is 0.31 nm for QD-0.3 ML, 0.31 nm for QD-0.7 ML, and 0.30 nm for QD-1 ML, which match the lattice spacing of (111) plane of cubic ZnS (d_111_ = 0.31 nm for zinc blende ZnS) [32]. By comparing it with CdSe (Figure S1), the spacing value of the (111) plane is 0.34 nm, which matches the bulk cubic zinc blende of CdSe. These results are evidence of the growth of the ZnS shell on the CdSe core.

#### XRD Patterns

**Fig. 2:**
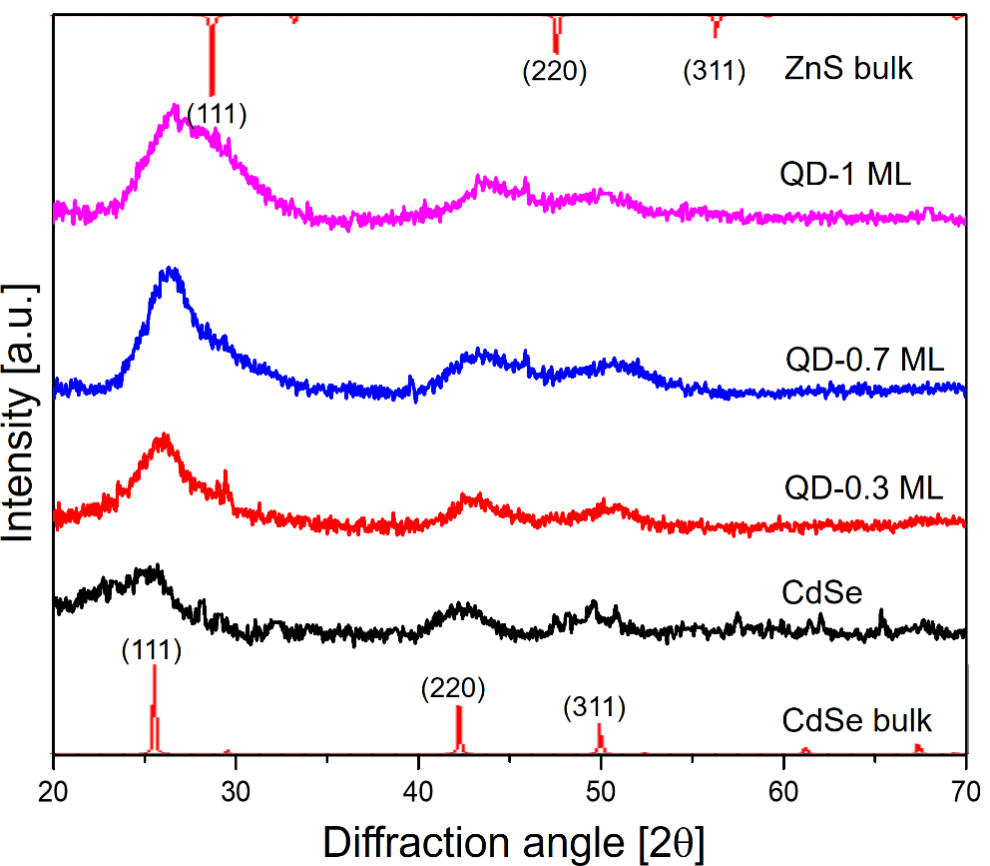
XRD patterns of CdSe (core) and CdSe/ZnS QDs with different shell thicknesses. The corresponding diffraction references of bulk CdSe (standard PDF card No. 03-065-2891) and bulk ZnS (standard PDF card No. 00-005-0566) are below and at the top, respectively

Figure 2 compares the X-ray powder diffraction (XRD) patterns of CdSe/ZnS samples with the CdSe pattern. For CdSe/ZnS samples, the diffraction peaks are centered around 25.5, 42.7, and 50.2 degrees, corresponding respectively to the (111), (220), and (311) lattice planes of cubic zinc blende CdSe (green peaks) [34]. Compared to CdSe, diffraction peaks of core/shell samples show more intense peaks and the characteristic shift to higher reflection angle positions due to the formation of ZnS shell [35, 36].

#### FT-IR Spectra

**Fig. 3:**
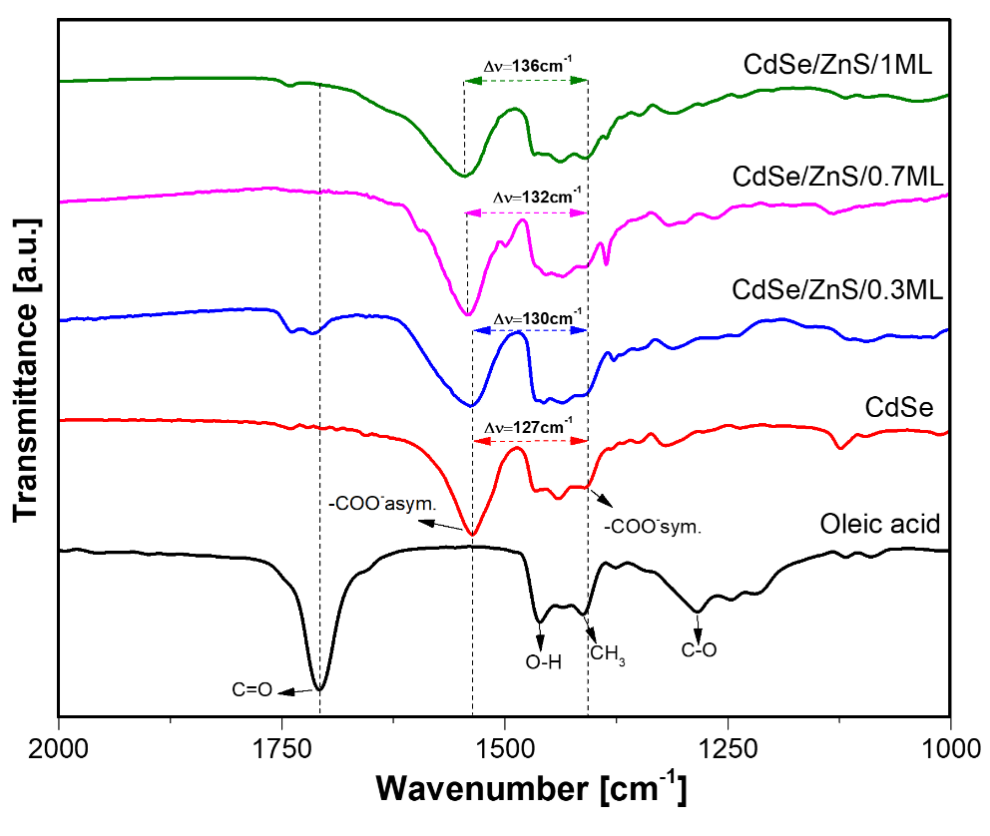
FT-IR spectra of oleic acid pure and the synthesized CdSe core and oleic acid-capped CdSe/ZnS core/shell with different shell thicknesses

Figure 3 shows FTIR spectra in the region 2000 –1000 cm^− 1^ of CdSe core and CdSe/ZnS QDs, which are compared with the oleic acid spectrum. The bands at 1460 cm^− 1^, 1412 cm^− 1^, and 1282 cm^− 1^, respectively, due to O-H, CH_3,_ and C-O groups of the pure oleic acid [37] are observed in all samples of QDs. However, for CdSe and CdSe/ZnS samples, the C-O band is shifted to higher wavenumbers, and the intense band at 1707 cm^− 1^, characteristic of C = O group of oleic acid, decreased significantly. FT-IR spectra of QDs samples show two bands around 1534 cm^− 1^ and 1407 cm^− 1^, which are respectively attributed to the asymmetric and symmetric stretching of the carboxylate anion (COO^−^), indicating a complexation between -COO^−^ group of oleic acid and a cation (Cd^2+^, Zn^2+^) of QDs surface. Separating asymmetric and symmetric carboxylate bands (Δν = ν_asym_COO^−^ – ν_sym_COO^−^) reveals the coordination mode between QDs surface and ligand. In the present case, the separation range is 127 cm^− 1^ and 136 cm^− 1^, suggesting a bidentate coordination mode [37, 38].

FT-IR spectra show a shift of the asymmetric band to higher wavenumbers as the shell thickness increases. This shift allows us to determine the cation affinity on QDs surface and -COO^−^ group. Since the amount of ZnS shell is different, the sites on QDs surface associated with the cation (Cd^2+^, Zn^2+^) must vary. CdSe is a reference sample with Cd^2+^ ions bound to oleic acid. QD-0.3 ML sample (with 0.3 monolayers) has Cd^2+^ and Zn^2+^ exposed on the QD surface, while QD-1 ML sample should have more Zn^2+^ ions exposed on surface to bind with oleic acid. According to the hard-soft acid-base (HSAB) theory [39], strong bonds are formed by electrostatic interaction between hard Lewis acid-base pairs and covalent interaction between soft pairs.

In contrast, a weak association is observed between members of opposite groups. In the present case, oleic acid, with oxygen-containing headgroups, is a hard base with more affinity to Zn^2+^ because it has a greater hardness than Cd^2+^ [16, 39]. Therefore, the stronger affinity of -COO^−^ group and Zn^2+^ of QDs surface produced a shift to higher wavenumbers of the asymmetric band. The observed shift indicates that QD-0.7 ML and QD-1 ML samples have more species of Zn^2+^ exposed on surface compared with QD-0.3 ML sample, which has more Cd^2+^ species (with a lower affinity to -COO^−^ group of ligand).

#### XPS Analysis

Figure S3 shows the typical XPS survey spectra of core and core/shell samples. Both spectra show the presence of Cd, Se, C, and O. CdSe/ZnS samples additionally show the presence of Zn and S.

**Fig. 4:**
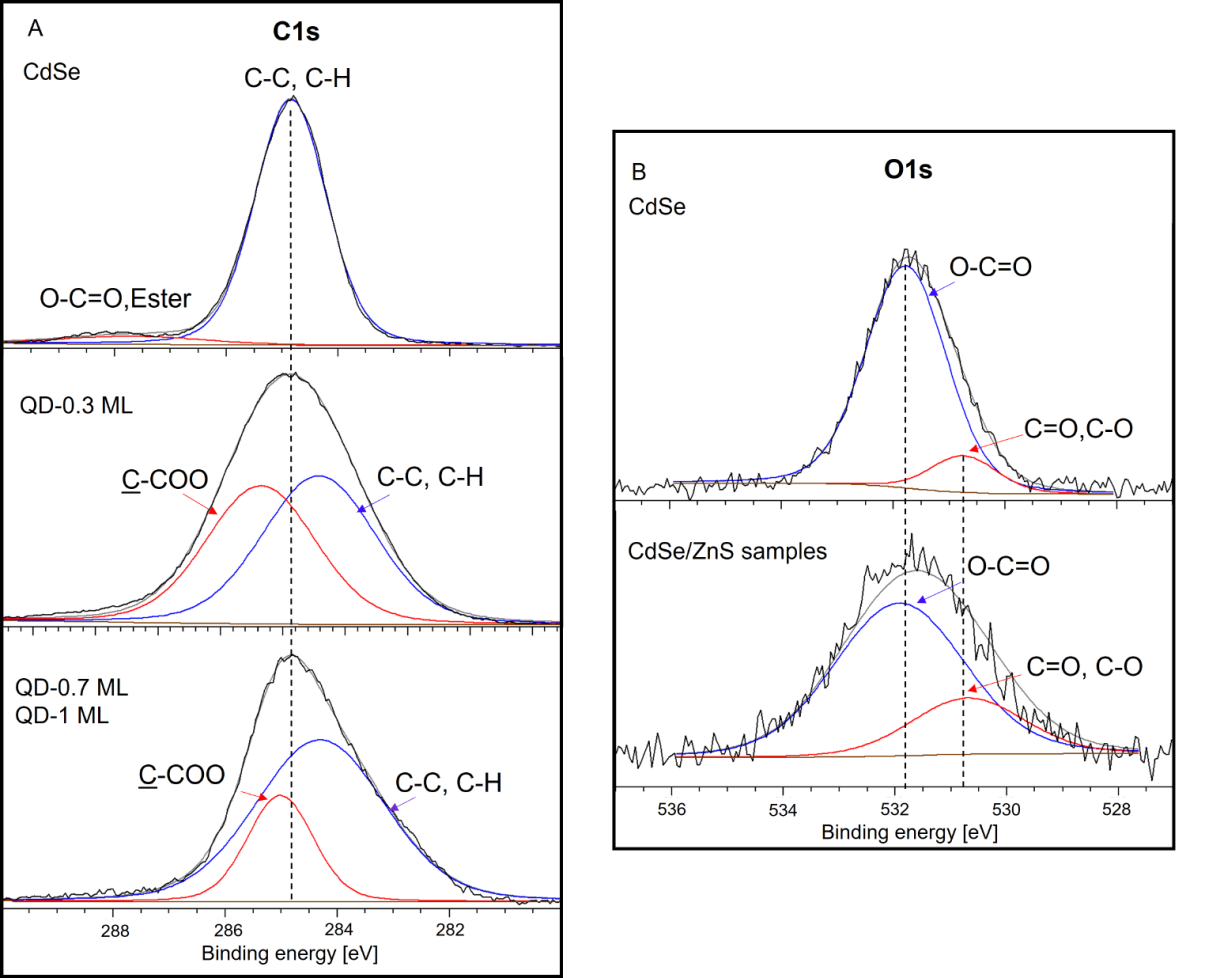
XPS spectra of CdSe and CdSe/ZnS samples: A) C1s, B) O1s

Figure 4 shows the high-resolution XPS spectrum of QDs samples. We compare the C1S and O1S spectra from oleic acid bound to CdSe and CdSe/ZnS surfaces. The C1s region is shown in Fig. 4A. The single peak of C1S of the CdSe core is deconvoluted into two components at 284.5 eV, associated with C-C and C-H bonds, and at 288.3 eV attributed to the bidentate carboxylate (–COO^−^) [40]. For core/shell samples, C1s peak is broader than the core’s spectrum, and the carboxylate peak around 288 eV is significantly reduced. QDs sample spectra are deconvoluted into two components: at 284.3 eV, attributed to C-C (or C-H) of oleic acid, and near 285.0 eV associated with the carbon adjacent to the carboxylic group, *C*-COO [41]. The O1s peak is shown in Fig. 4B, and a few changes between core and core/shell samples could be appreciated. Core/shell samples show a broader noisy peak, which is deconvoluted into two components at 530.7 eV, attributed to C–O and C = O of monodentate carboxylate, and at 532 eV assigned to bidentate carboxylate. Results demonstrate the chemical bonding between oleic acid and QDs surface.

**Fig. 5:**
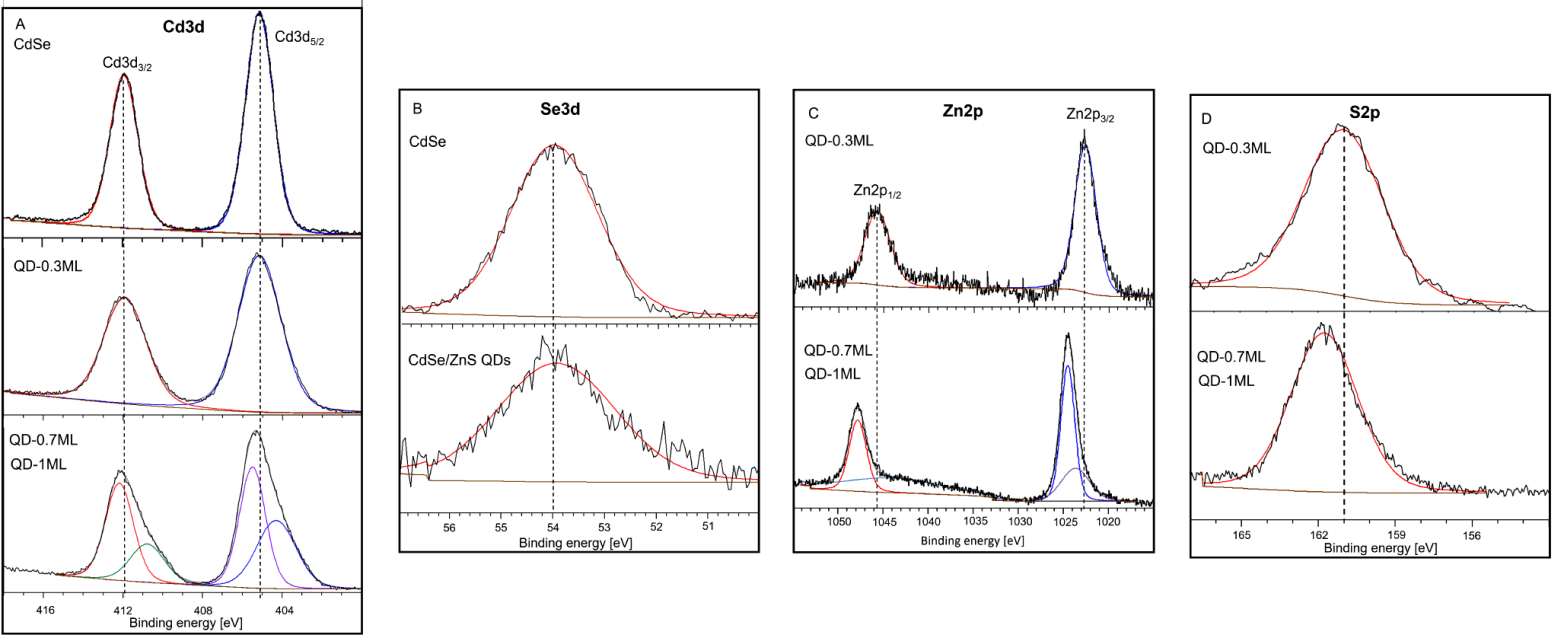
XPS spectra of CdSe and CdSe/ZnS samples: A) Cd3d, B) Se3d, C) Zn2p, and D) S2p

Figure 5 shows Cd3d, Se3d, Zn2p, and S2p spectra of core and core/shell samples. Figure 5A shows the Cd3d region. For CdSe core, the characteristic doublet of Cd^2+^ is seen at 405.1 eV and 411.9 eV, which is attributed to Cd3d_5/2_ and Cd3d_3/2,_ respectively. For the QD-0.3 ML sample, the Cd3d spectrum remained almost unchanged (compared with core). For samples with a thicker shell (QD-0.7 ML and QD-1 ML), Cd3d doublet is moved to slightly higher binding energies. Additionally, the doublet is deconvoluted into two components, indicating different environments of Cd^2+^ on surface, e.g., Cd^2+^ binding to Se^2−^ (or COO^−^) and underpassivated Cd^2+^ [42].

The Se3d peak for the core sample is observed at 54.1 eV (Fig. 5B), a characteristic of metal selenides [43]. The Se3d spectra for core/shell samples are very similar to those obtained for CdSe core; however, the signal is less intense and noisier due to the shell on core.

The Zn2p region of core/shell samples is shown in Fig. 5C. QD-0.3 ML sample shows the Zn2p peak resolved into two splitting peaks at 1045.7 eV and 1022.6 eV, corresponding to Zn2p_1/2_ and Zn2p_3/2_, respectively. The separation between them is 23.1 eV, characteristic of Zn^2+^ species [44]. QDs with a thicker shell (QD-0.7 ML and QD-1 ML) have similar profiles. The Zn2p doublet is shifted to higher binding energies. The peaks are asymmetrical and broader compared with QD-0.3 ML sample. These changes reveal the presence of various Zn^2+^ species, such as Zn^2+^ bound to S^2−^ and oleic acid and the under-passivated Zn^2+^. These Zn^2+^ species could be more evident in the samples with a thicker shell since the molar ratios of Zn and S precursors were more equivalent than QD-0.3 ML.

The S2p region is shown in Fig. 5D. For QD-0.3 ML, the broad peak centered at 161.0 eV could be due to different sulfur species (S^2−^ bonded to Zn^2+^ or underpassivated S^2−^) generated from S^2−^-rich solutions. In samples with the thicker shell, S2p peak is sharper and shifted to higher binding energies, indicating that when ZnS shell grows up from Zn and S concentrations with a molar equivalence, the under-passivated S^2−^ species are diminished.

Considering the FT-IR and XPS results, the growth of a ZnS shell by varying [S]/[Zn] molar ratio in the precursor solutions generated underpassivated ionic species (Cd^2+^, Zn^2+^, and S^2−^), which could be evidenced by the broader and shifted peaks in the XPS spectra, particularly in the Cd3d, Zn2p, S2p regions. QD-0.3ML has more underpassivated S^2−^ species derived from S^2−^-rich solutions. Samples with a thicker shell ([S]/[Zn] molar ratio was 2/1 and 1/1) are characterized by having more underpassivated Zn^2+^ species. As was observed in the FT-IR spectra, for instance, QD-1 ML with more Zn^2+^ species in the QDs surface produced a shift that was more significant in the band related to bond -COO^−^-Zn^2+^.

### Optical Characterization

**Fig. 6:**
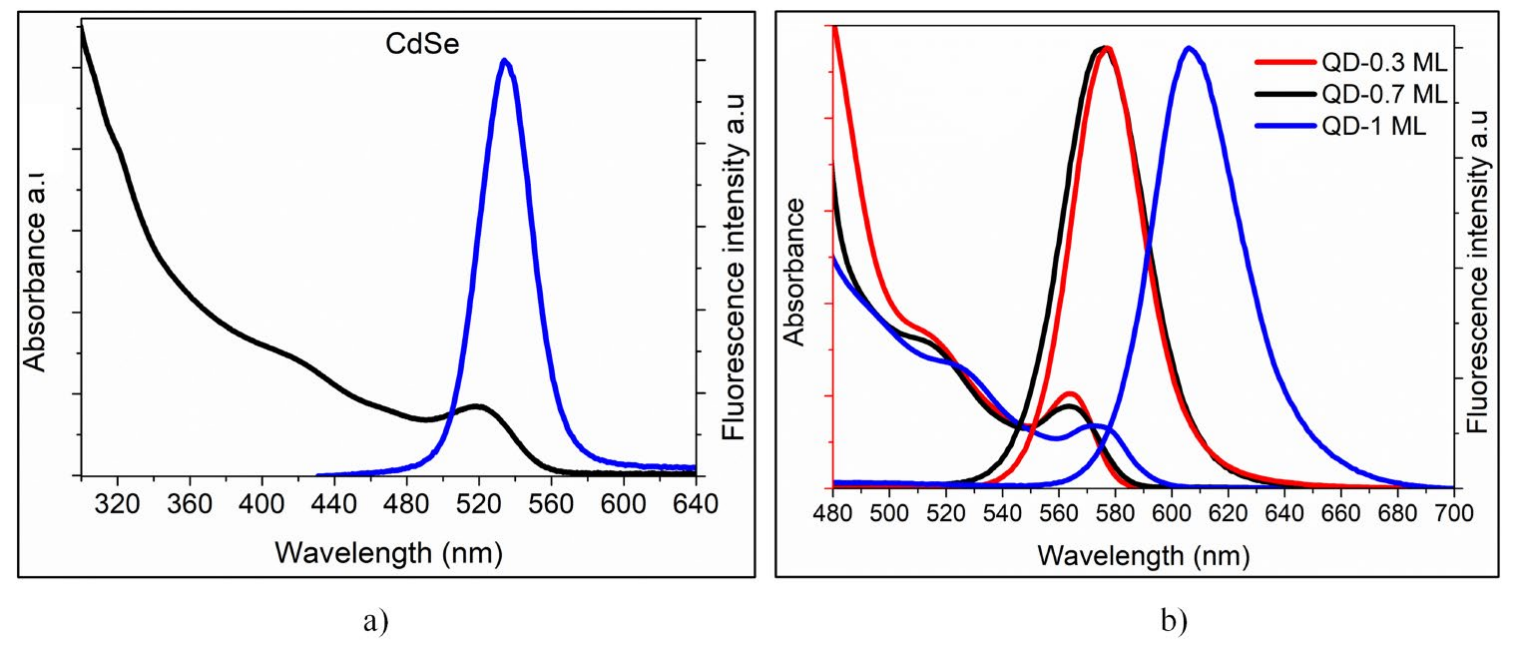
Absorption and PL spectra of CdSe and CdSe/ZnS nanocrystals at room temperature, λ_exc_: 380 nm

Figure 6; Table 2 compare the main characteristics of the absorption and emission spectra of CdSe core and CdSe/ZnS samples. CdSe/ZnS samples show the typical red-shift in the first exciton absorption peak and the emission maximum regarding CdSe. The PLQY enhanced from 0.17 for CdSe to 0.54 for core/shell samples. This enhancement of PLQY is a typical result of the growth of ZnS shell on CdSe core [45]. Literature has demonstrated that ZnS shell passivates defects of core surface, increasing PLQY [21, 46]. However, PLQY is affected when many ZnS monolayers grow on the core [46]. In this work, a relationship between QY and the thickness of the ZnS shell is not observed since QD-0.7 ML has a higher PLQY (0.54), and the sample with one formed monolayer of ZnS shows a reduction of PLQY (0.47). We believe that the variation of the molar ratio of precursors could introduce trap states that affected the PLQY.

**Table 2:** Optical properties of CdSe and CdSe/ZnS

QDs	First absorption peak [nm]	Emission maximum [nm]	Stokes shift [eV]	Emission bandwidth (FWHM) [nm]	PL QY
CdSe	518	533	0.050	35 ± 0.1	0.17
QD-0.3 ML	563	576	0.040	31 ± 0.2	0.46
QD-0.7ML	564	578	0.047	34 ± 0.1	0.54
QD-1 ML	581	606	0.070	37 ± 0.1	0.47

**Fig. 7:**
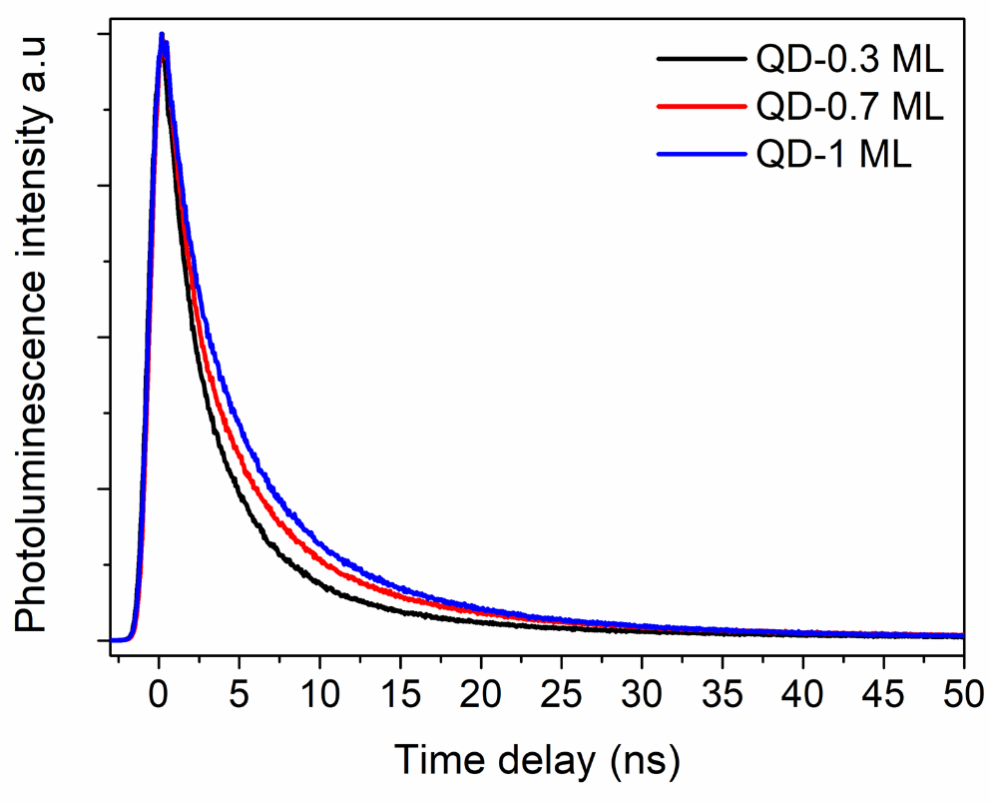
Photoluminescence decay curves of CdSe/ZnS core/shell QDs with different shell thickness dissolved in chloroform, λ_em_: 590 nm

Photoluminescence lifetime measurements were performed to understand the role of traps associated with [S]/[Zn] molar ratio and the exciton dynamics (Fig. 7). Nonexponential kinetics showed the PL decay curves of core and core/shell QDs. Still, they could be mathematically fit by multiple exponentials (Eq. 4), with relative amplitudes *A*_*i*_ and time constants τ_i_. The average fluorescence lifetime (*τ*_*FL*_) is calculated with Eq. 5.

4 $$\eqalign{ I\left( t \right) = & {I_0} + {A_1}{e^{\left( { - {t \over {{\tau _1}}}} \right)}} + {A_2}{e^{\left( { - {t \over {{\tau _2}}}} \right)}}{A_3}{e^{\left( { - {t \over {{\tau _3}}}} \right)}} \cr & + {A_4}{e^{\left( { - {t \over {{\tau _4}}}} \right)}} \cr}$$

5 $$\left\langle {{\tau _{FL}}} \right\rangle = \sum\nolimits_{i = 4}^4 {{A_i}{\tau _i}} /\sum\nolimits_{i = 4}^4 {{A_i}}$$

Table 3 shows the average fluorescence lifetimes (*τ*_*FL*_). A dependence of *τ*_*FL*_ values with shell thickness is observed. The ZnS shell passivates the non-radiative trap states on the CdSe core, making the core more photostable and less affected by non-radiative recombination pathways [22, 46–48]. No direct correlation was found between the average fluorescence lifetime and QY values, due probably to the trapping sites introduced in the nanocrystal structure during the synthesis process. We recall that QD-0.3 ML sample with the thinner shell was prepared from a solution rich in S^2−^, which generated underpassivated S^2−^ species on QDs surface. The anions (underpassivated S^2−^ species) are known for their role as hole traps [49, 50], affecting PLQY for QD-0.3 ML sample. By contrast, the sample with one monolayer of ZnS (QD-1 ML), obtained from an equal ratio of [S]/[Zn] precursors, is characterized by having underpassivated S^2−^ and Zn^2+^ species. According to previous investigations, PLQY increases up to a specific shell thickness (1.25 ML − 1.3 ML of ZnS [21]). After any further increase in the shell thickness, PL decreases due to the introduction of non-radiative recombination sites [46, 51, 52]. In the present investigation, the reduction of PLQY in QD-1 ML could be due to underpassivated Zn^2+^ and S^2−^ species acting as electron and hole traps [53], respectively decreasing PL.

**Table 3:** Luminescence lifetimes measured by time-correlated single-photon counting (TCSPC) of CdSe/ZnS QDs

QDs	τ_FL_(ns)	χ^2^
QD-0.3 ML	3.29	1.49
QD-0.7 ML	4.34	1.14
QD-1 ML	4.82	1.05

#### Effect of Hg^2+^ Ions on Luminescence Properties

**Fig. 8:**
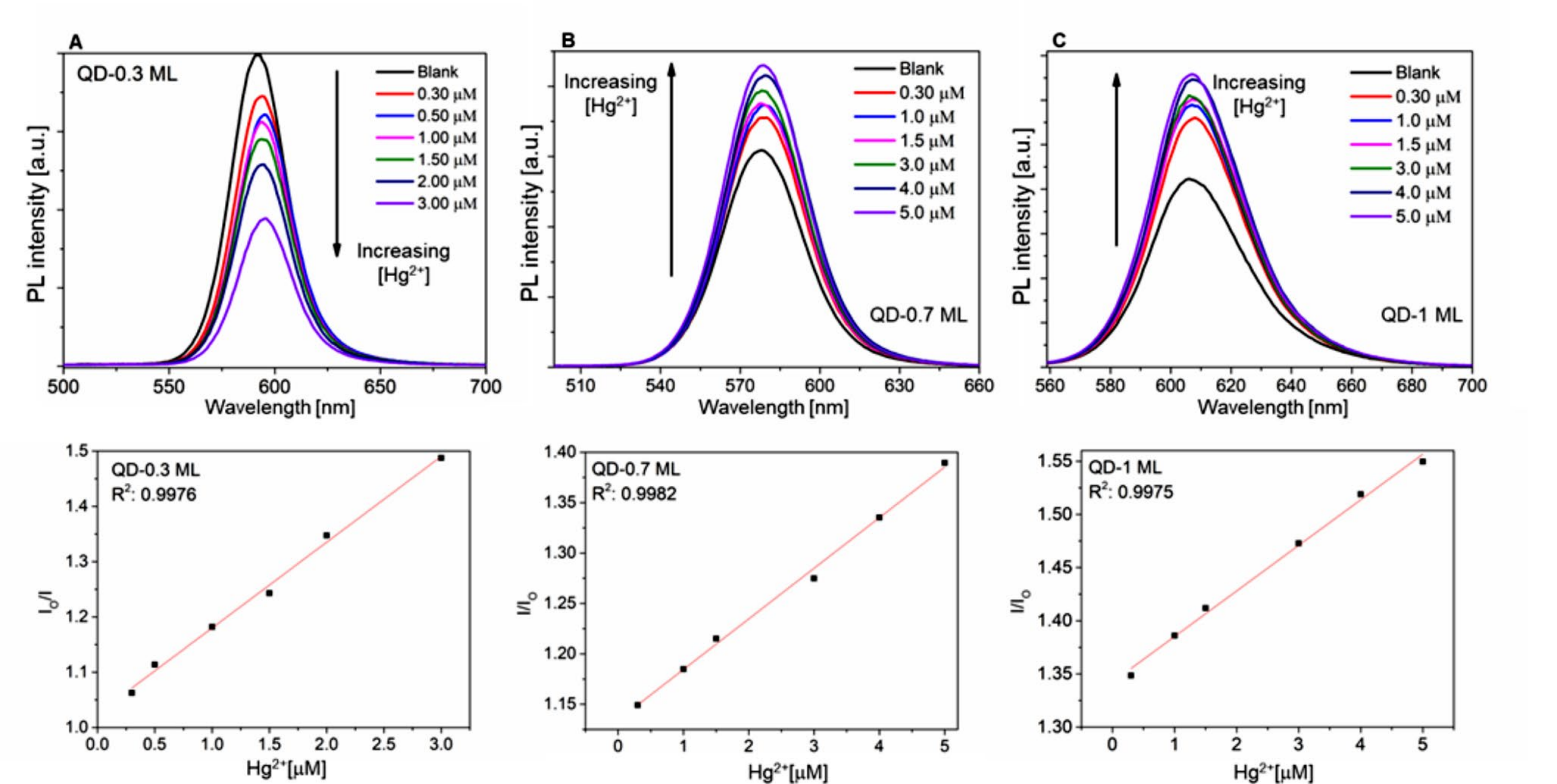
Effect of Hg^2+^ on PL spectra of oleic acid-capped CdSe/ZnS QDs. (A) PL quenching using QD-0.3 ML sample, (B) PL enhancing using QD-0.7 ML sample, and (C) PL enhancing using QD-1 ML sample. At the bottom the Stern-Volmer relationship between fluorescence intensity of CdSe/ZnS QDs and Hg^2+^ concentration

The changes in the PL spectra of CdSe/ZnS QDs samples induced by Hg^2+^ ions were studied in a homogeneous mixture containing QDs dissolved in chloroform and Hg^2+^ ions dissolved in water. Results show that the PL intensity could be quenched or enhanced by Hg^2+^ ions. The two opposite effects are shown in Fig. 8 and depend on CdSe/ZnS surface properties (thickness shell and surface traps). First, we presented the progressive fluorescence quenching by increasing the Hg^2+^ concentration for QD-0.3 ML sample (Fig. 8A), accompanied by a shift of 3 nm of maximum emission wavelength. FL quenching by Hg^2+^ could be explained by the metal cation exchange reaction that produces the displacement of Cd^2+^ or Zn^2+^ in the lattice by Hg^2+^ ions generating HgSe (or HgS) particles on surface. This cation exchange is due to the lower solubility constant (K_SP_) of HgSe or HgS compared with K_SP_ CdSe or ZnS being favored thermodynamically, as discussed previously in several reports [54].

The PL quenching by increasing the Hg^2+^ concentration in the range of 0.3-3.0 µM is evaluated in terms of Stern-Volmer equation (Eq. 6):

6 $${I_o}/I = {\rm{ }}1 + {K_{sv}}\left[ {H{g^{2 + }}} \right]$$

*I*_*o*_ and *I* are the fluorescence intensities at a constant wavelength in a mercury ion-free solution and a given mercury ion concentration, respectively. [Hg^2+^] is the concentration of Hg^2+^, and *K*_sv_ is the Stern-Volmer FL quenching constant. A good lineality is obtained with a correlation coefficient (R^2^) equal to 0.9976 (bottom of Fig. 8A). The limit of detection (LOD), calculated with equation LOD = 3σ/*k*, where σ is the standard deviation of the y-intercept of the regression line, and *k* is the slope of the calibration graph, is found to be 0.0112 µM (11.2 nM). This value is lower than previously reported using oleic acid-capped CdSe/ZnS in a heterogeneous mixture that produces PL quenching induced by Hg^2+^ [30].

Figure 8B and C show the enhancement of PL intensity of the QD-0.7 ML and QD-1 ML samples in the presence of Hg^2+^ in the concentration range from 0.3 µM to 5.0 µM. The enhancement of PL (*I/I*_*o*_) of these two QDs is evaluated by the equation: *I/I*_*o*_ = 1+*K*_sv_[Hg^2+^], where *K*_sv_ is the Stern-Volmer constant for the enhanced PL. A good linearity is obtained for both samples (see bottom of Fig. 7B and C): R^2^ is 0.9982, and LOD is 0.00898 µM (8.98 nM) for QD-0.7 ML sample. R^2^ is 0.9975, and LOD is 0.0107 µM (10.7 nM) for QD-1 ML sample. The enhancement of PL by CdSe/ZnS with oleic acid as a capping agent is observed for the first time. The enhanced PL of QDs induced by Hg^2+^ [31] and other metal ions [55] has been reported in other investigations. Results reveal the shell’s role in detecting Hg^2+^. A thicker shell (than 0.3 ML) protects the core from the exchange reaction with Hg^2+^, providing more stability and brightness. Furthermore, thicker shell samples (QD-0.7 ML and QD-1ML) have more underpassivated Zn^2+^ species on the surface, which are more exposed for the exchange with Hg^2+^ than Cd^2+^ species from core. These results show a significant improvement in the emission properties of oleic acid-capped CdSe/ZnS and an improved capacity to detect Hg^2+^ with lower LOD values.

**Fig. 9:**
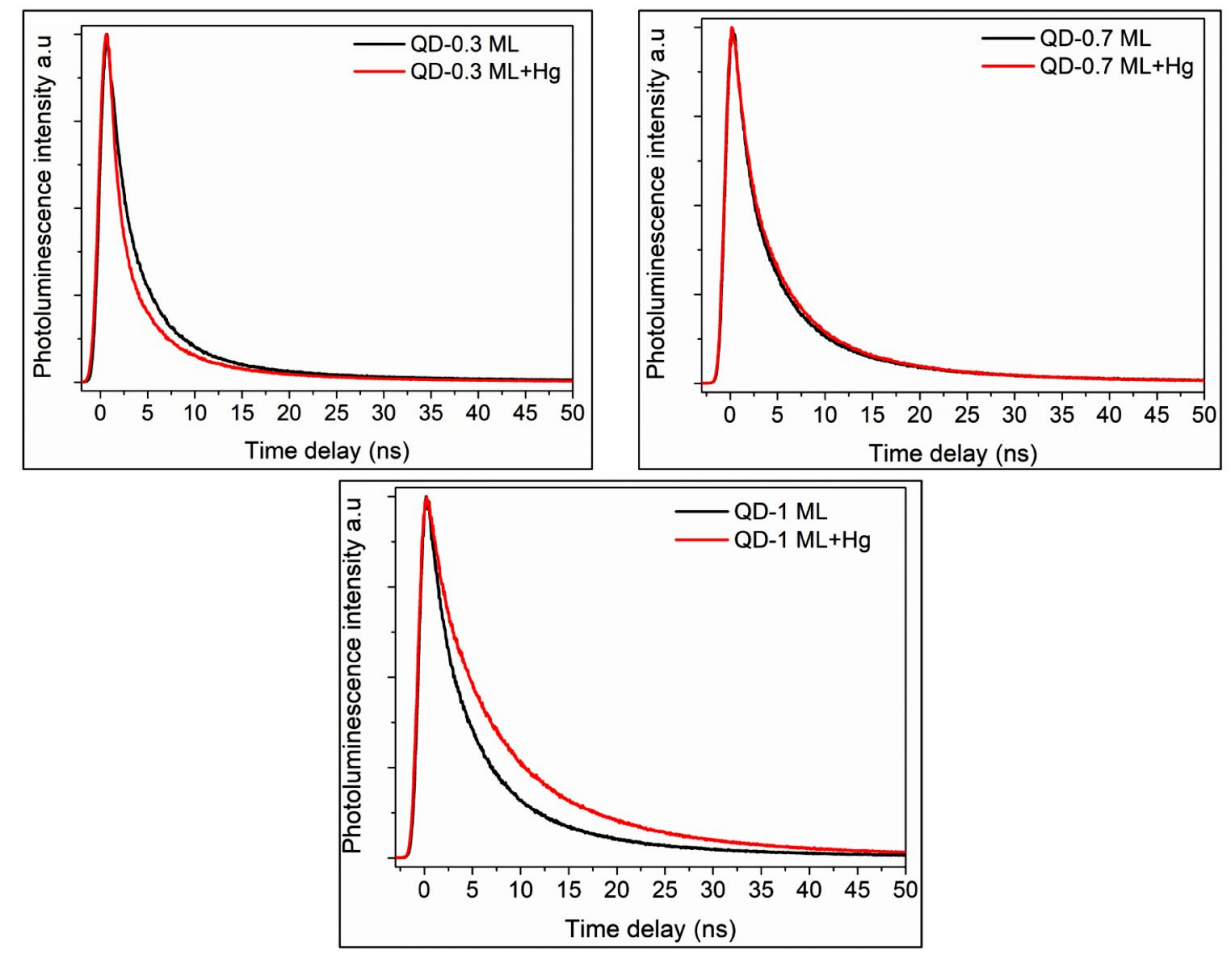
Photoluminiscence decay curves of CdSe/ZnS QDs with different shell thicknesses before and after adding Hg^2+^ ions (5 μM). λem: 590 nm. Solvent: chloroform/ethanol (1:1)

The influence of Hg^2+^ on the PL lifetimes was investigated. Figure 9 shows the PL decay curves for CdSe/ZnS samples mixed with an Hg^2+^ aqueous solution. Table 4 gives the average fluorescence lifetimes, showing that the decays are very sensitive to the surface states of QDs. When Hg^2+^ is present, longer lifetimes occur for samples with a thicker shell (0.7 and 1.0 ML). The increased PL of CdSe/ZnS QDs by mercury ions could be attributed to the Zn^2+^-to-Hg^2+^ cation exchange in the ZnS shell, which favors the separation of electrons and holes, reducing the recombination rate [31].

**Table 4:** Luminescence lifetime measured by TCSPC of CdSe/ZnS QDs in presence of Hg^2+^ solution

QDs + [Hg^2+^]	τ_FL_(ns)	χ^2^
QD-0.30 ML	2.81	1.10
QD-0.7 ML	4.43	1.12
QD-1.0 ML	5.00	1.06

QD-0.3 ML mixed with Hg^2+^ produces shorter average fluorescence lifetimes, corroborating the effect of these cations to PL detriment. Similar sensing systems have also reported shorter FL lifetimes, accompanied by FL quenching in presence of metal ions [56, 57]. As we explained, FL quenching by Hg^2+^ is derived by the metal cation exchange reaction with Cd^2+^ (from core) or Zn^2+^ (from shell), generating HgSe and HgS particles that quench FL. In addition, the underpassivated S^2−^ species in the QD-0.3 ML sample bind to Hg^2+^, producing HgS and HgSe.

#### Effect of Other Metal Ions

**Fig. 10:**
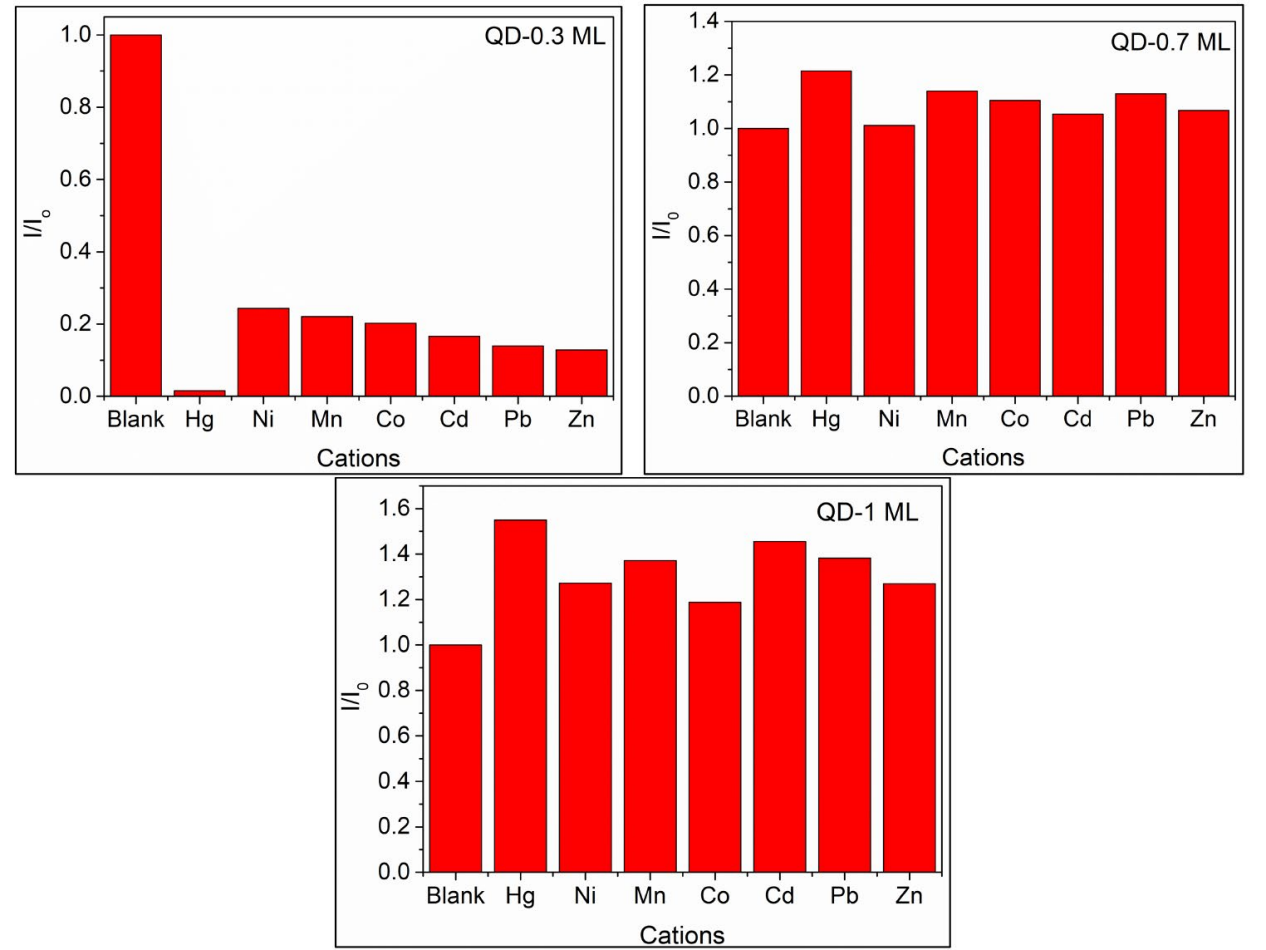
Relative photoluminescence response of CdSe/ZnS QDs with different shell thicknesses in the presence of Hg^2+^ and other metal transition ions

The effect of other metal transition cations on the fluorescence of CdSe/ZnS quantum dots (QD-0.3 ML, QD-0.7 ML, and QD-1 ML) was performed in homogeneous mixtures. A solution of QDs in chloroform/ethanol (1/1 v/v) was mixed with aqueous solutions of metal chloride salts (Co^2+^, Mn^2+^, Ni^2+^, Zn^2+^, Pb^2+^, Cd^2+^ ) (Fig. 10). For QD-0.3 ML sample, the reduction of fluorescence intensity was observed with all the evaluated cations; however, Hg^2+^ reduced 100% fluorescence quenching. This result could be explained by the cation exchange reaction between Hg^2+^ ions and Se^2−^ and S^2−^ anions. Based on the *K*_*sp*_ values of HgSe (4.0 × 10^− 59^) and HgS (1.6 × 10^− 52^), which are lower than other ions [16, 39], a better ability of Hg^2+^ to PL quench is expected.

In the QD-0.7 ML and QD-1 ML samples, all the evaluated ions produce fluorescence *turn-on* (Fig. 10). However, the turn-on effect of Hg^2+^ induced is higher [16, 39]. The role of the shell is essential to protect the core and avoid cation exchange reactions with foreign metal ions. We demonstrated that cations can passivate the surface of the QDs, eliminating some trapping sites and thus increasing the photoluminescence.

## Conclusions

Three samples of oleic acid-capped CdSe/ZnS QDs with different shell thicknesses (0.3 ML, 0.7 ML, and 1.0 ML) were obtained by varying the [S]/[Zn] molar ratio of shell precursors. Characterization measurements of their optical and structural properties revealed differences in chemical speciation. Excess of S^2−^ precursor generated underpassivated S^2−^ species in the sample with the thinner shell. In contrast, in samples with a thicker shell (obtained with a [S]/[Zn] molar ratio very close), the underpassivated Zn^2+^ species were primarily formed. Underpassivated S^2−^ and Zn^2+^ species were related to hole and electron trapping sites, respectively, which affect PL.

The ability of CdSe/ZnS QDs samples to detect Hg^2+^ by PL quenching and enhancing was studied. These oppositive mechanisms could be tuned due to changes in the thickness and chemical speciation in core/shell QDs. PL quenching is produced for the sample with the thinner shell due probably to the cation exchange of Hg^2+^ by Cd^2+^ (from core) affecting PL. Samples with a thicker shell (with 0.7 and 1.0 ML) protect the core of the unwanted cation exchange reaction, producing longer fluorescence lifetimes and better PL. In addition, Hg^2+^ ions contribute to the passivation of traps. In detecting Hg^2+^, the lower LOD value (8.98 nM) was obtained with the CdSe/ZnS sample with 0.7 ML. However, other foreign cations also achieved increased PL; therefore, the selectivity is affected. Results show that core/shell QDs are promising for detecting Hg^2+^ ions, and tuning shell properties is a determinant factor in the sensitivity and selectivity of the application.

## Electronic Supplementary Material

Below is the link to the electronic supplementary material.

Supplementary Material 1

## Data Availability

All data generated or analyzed during this study are included in this manuscript and its supplementary information file.

## References

[1] Reiss P, Protière M, Li L (2009) Core/shell semiconductor nanocrystals. Small 5:154–168. 10.1002/smll.200800841 19153991 10.1002/smll.200800841

[2] Vasudevan D, Gaddam RR, Trinchi A, Cole I (2015) Core-Shell Quantum dots: Properties and Applications. 10.1016/j.jallcom.2015.02.102. J Alloys Compd

[3] Fernández-delgado N, Herrera M, Tavabi AH et al (2018) Applied Surface Science Structural and chemical characterization of CdSe-ZnS core-shell quantum dots. Appl Surf Sci 457:93–97. 10.1016/j.apsusc.2018.06.149

[4] Jin LH, Han CS (2014) Ultrasensitive and selective fluorimetric detection of copper ions using thiosulfate-involved quantum dots. Anal Chem 86:7209–7213. 10.1021/ac501515f 24981053 10.1021/ac501515f

[5] Zhou J, Liu Y, Tang J, Tang W (2017) Surface ligands engineering of semiconductor quantum dots for chemosensory and biological applications. Biochem Pharmacol 20:360–376. 10.1016/j.mattod.2017.02.006

[6] Lou Y, Zhu J (2014) Metal ions optical sensing by semiconductor quantum dots. J Mater Chem C Mater 2:585–772. 10.1039/c3tc31937g

[7] Ren J, Chen HL, Ren CL et al (2010) L-cysteine capped CdSe as sensitive sensor for detection of trace lead ion in aqueous solution. Mater Res Innovations 14:133–137. 10.1179/143307510X12639910071476

[8] Liang JG, Ai XP, He ZK, Pang DW (2004) Functionalized CdSe quantum clots as selective silver ion chemodosimeter. Analyst 129:619–622. 10.1039/b317044f 15213829 10.1039/b317044f

[9] Fernández-Argüelles MT, Wei JJ, Costa-Fernández JM et al (2005) Surface-modified CdSe quantum dots for the sensitive and selective determination of Cu(II) in aqueous solutions by luminescent measurements. Anal Chim Acta 549:20–25. 10.1016/j.aca.2005.06.013

[10] Li H, Zhang Y, Wang X, Gao Z (2008) A luminescent nanosensor for hg(II) based on functionalized CdSe/ZnS quantum dots. Microchim Acta 160:119–123. 10.1007/s00604-007-0816-x

[11] Wu P, Yan XP (2010) A simple chemical etching strategy to generate ion-imprinted sites on the surface of quantum dots for selective fluorescence turn-on detecting of metal ions. Chem Commun 46:7046–7048. 10.1039/c0cc01762k 10.1039/c0cc01762k 20730211

[12] Pendyala NB, Koteswara Rao KSR (2009) Efficient hg and ag ion detection with luminescent PbS quantum dots grown in poly vinyl alcohol and capped with mercaptoethanol. Colloids Surf Physicochem Eng Asp 339:43–47. 10.1016/j.colsurfa.2009.01.013

[13] Chen JL, Zhu CQ (2005) Functionalized cadmium sulfide quantum dots as fluorescence probe for silver ion determination. Anal Chim Acta 546:147–153. 10.1016/j.aca.2005.05.006

[14] Chern M, Kays JC, Bhuckory S, Dennis AM (2019) Sensing with photoluminescent semiconductor quantum dots. Methods Appl Fluoresc 7. 10.1088/2050-6120/aaf6f8 10.1088/2050-6120/aaf6f8 PMC7233465 30530939

[15] Demchenko AP (2015) Introduction to Fluorescence Sensing. Kiev-Ukraine

[16] De Trizio L, Manna L (2016) Forging colloidal nanostructures via cation exchange reactions. Chem Rev 116:10852–10887 26891471 10.1021/acs.chemrev.5b00739 PMC5043423

[17] Rodrigues SSM, Ribeiro DSM, Soares JX et al (2017) Application of nanocrystalline CdTe quantum dots in chemical analysis: implementation of chemo-sensing schemes based on analyte-triggered photoluminescence modulation. Coord Chem Rev 330:127–143

[18] Hartley CL, Kessler ML, Dempsey JL (2021) Molecular-level insight into Semiconductor Nanocrystal surfaces. J Am Chem Soc 143:1251–1266. 10.1021/jacs.0c10658 33442974 10.1021/jacs.0c10658

[19] Houtepen AJ, Hens Z, Owen JS, Infante I (2017) On the origin of Surface traps in Colloidal II-VI Semiconductor nanocrystals. Chem Mater 29:752–761. 10.1021/acs.chemmater.6b04648

[20] Ren C, Hao J, Chen H et al (2015) Prepare core-multishell CdSe/ZnS nanocrystals with pure color and controlled emission by tri-n-octylphosphine-assisted method. Appl Surf Sci 353:480–488. 10.1016/j.apsusc.2015.06.149

[21] Dabbousi BO, Rodriguez-Viejo J, Mikulec FV et al (1997) (CdSe)ZnS core – Shell Quantum dots: synthesis and characterization of a size series of highly luminescent nanocrystallites. J Phys Chem B 101:9463–9475. 10.1021/jp971091y

[22] Mathew S, Bhardwaj BS, Saran AD et al (2015) Effect of ZnS shell on optical properties of CdSe-ZnS core-shell quantum dots. Opt Mater (Amst) 39:46–51. 10.1016/j.optmat.2014.10.061

[23] Reiss P, Pron A (2002) Highly luminescent CdSe / ZnSe Core / Shell Nanocrystals of low size dispersion. 21–24

[24] Lien VTK, Tan PM, Hien NT et al (2019) Tunable photoluminescent Cu-doped CdS/ZnSe type-II core/shell quantum dots. J Lumin 215:116627. 10.1016/J.JLUMIN.2019.116627

[25] Sanmartín-Matalobos J, Bermejo-Barrera P, Aboal-Somoza M et al (2022) Semiconductor Quantum Dots as Target Analytes: Properties, Surface Chemistry and Detection. Nanomaterials 2022, Vol 12, Page 2501 12:2501. 10.3390/NANO12142501 10.3390/nano12142501 PMC9318497 35889725

[26] Vinayakan R, Shanmugapriya T, Nair PV et al (2007) An approach for optimizing the shell thickness of core - Shell quantum dots using photoinduced charge transfer. J Phys Chem C 111:10146–10149. 10.1021/jp072823h

[27] Pisheh HS, Gheshlaghi N, Ünlü H (2017) The effects of strain and spacer layer in CdSe/CdS/ZnS and CdSe/ZnS/CdS core/shell quantum dots. Phys E Low Dimens Syst Nanostruct 85:334–339. 10.1016/j.physe.2016.07.007

[28] Speranskaya ES, Goftman VV, Goryacheva IY (2013) Preparation of water soluble zinc-blende CdSe/ZnS quantum dots. Nanotechnol Russ 8:129–135. 10.1134/S1995078013010163

[29] Baranov V, Rakovich YP, Donegan F et al (2003) Effect of ZnS shell thickness on the phonon spectra in CdSe quantum dots. Phys Rev B Condens Matter Mater Phys 68. 10.1103/PhysRevB.68.165306

[30] Granados-Oliveros G, Pineros BSG, Calderon FGO (2022) CdSe/ZnS quantum dots capped with oleic acid and L-glutathione: structural properties and application in detection of Hg2+. J Mol Struct 1254:132293. 10.1016/J.MOLSTRUC.2021.132293

[31] Wang H, Song D, Zhou Y et al (2021) Fluorescence enhancement of CdSe/ZnS quantum dots induced by mercury ions and its applications to the on-site sensitive detection of mercury ions. Microchim Acta 188:1–9. 10.1007/S00604-021-04871-5/METRICS 10.1007/s00604-021-04871-5 34052914

[32] Hao J, Liu H, Miao J et al (2019) A facile route to synthesize CdSe/ZnS thick-shell quantum dots with precisely controlled green emission properties: towards QDs based LED applications. Sci Rep 9. 10.1038/s41598-019-48469-7 10.1038/s41598-019-48469-7 PMC6700096 31427624

[33] Grabolle M, Spieles M, Lesnyak V et al (2009) Determination of the Fluorescence Quantum Yield of Quantum Dots. Suitable Procedures Achievable Uncertainties 81:6285–6294. 10.1021/ac900308v

[34] Gao Y, Yin PG (2017) Synthesis of cubic CdSe nanocrystals and their spectral properties. Nanomaterials Nanatechnol 7. 10.1177/1847980417701747

[35] Botao Ji SKISSRUB (2020) ZnSe-ZnS Core-Shell Quantum Dots with Superior Optical. Nano Lett 20:2387–2395. 10.1021/acs.nanolett.9b05020 32134676 10.1021/acs.nanolett.9b05020 PMC7467768

[36] Hien NT, Vinh ND, Thanh LD et al (2019) Synthesis, characterization and the photoinduced electron-transfer energetics of CdTe/CdSe type-II core/shell quantum dots. J Luminiscence. 10.1016/j.jlumin.2019.116822

[37] Premaratne W, Priyadarshana W, Gunawardena S et al (2013) SYNTHESIS OF NANOSILICA FROM PADDY HUSK ASH AND THEIR SURFACE FUNCTIONALIZATION

[38] Kim KM, Jeon JH, Kim YY et al (2015) Effects of ligand exchanged CdSe quantum dot interlayer for inverted organic solar cells. Org Electron 25:44–49. 10.1016/j.orgel.2015.05.040

[39] Pearson RG, Busch DH (1963) Hard and soft acids and bases. J Am Chem Soc 84:3533–3539

[40] Zhang N, Xie J, Varadan VK (2002) Functionalization of carbon nanotubes by potassium permanganate assisted with phase transfer catalyst. Smart Mater Struct 11:962–965. 10.1088/0964-1726/11/6/318

[41] Watts JF, Wolstenholme J (2003) An Introduction to Surface Analysis by XPS and AES. An Introduction to Surface Analysis by XPS and AES. 10.1002/0470867930

[42] Vale BRC, Mourão RS, Bettini J et al (2019) Ligand induced switching of the band alignment in aqueous synthesized CdTe/CdS core/shell nanocrystals. Sci Rep 9:1–12. 10.1038/s41598-019-44787-y 31171820 10.1038/s41598-019-44787-y PMC6554334

[43] Bowen Katari JE, Colvin VL, Alivisatos AP (1994) X-ray Photoelectron Spectroscopy of CdSe nanocrystals with applications. to Studies of the Nanocrystal Surface

[44] Granada-Ramirez DA, Arias-Cerón JS, Gómez-Herrera ML et al (2019) Effect of the indium myristate precursor concentration on the structural, optical, chemical surface, and electronic properties of InP quantum dots passivated with ZnS. J Mater Sci: Mater Electron 30:4885–4894. 10.1007/s10854-019-00783-6

[45] Gómez-Pineros BS, Granados-Oliveros G (2018) Synthesis and characterization of optic properties of CdSe and CdSe/ZnS quantum dots. Rev Colomb Quim 47. 10.15446/rev.colomb.quim.v47n1.61067

[46] Rajapaksha RD, Ranasinghe MI (2017) The shell thickness and surface passivation dependence of fluorescence decay kinetics in CdSe/ZnS core-shell and CdSe core colloidal quantum dots. J Lumin 192:860–866. 10.1016/j.jlumin.2017.08.024

[47] Sambur JB, Parkinson BA (2010) CdSe/ZnS core/shell quantum dot sensitization of low index tio2 single crystal surfaces. J Am Chem Soc 132:2130–2131. 10.1021/ja9098577 20121191 10.1021/ja9098577

[48] Li J, Zheng H, Zheng Z et al (2022) Synthesis of CdSe and CdSe/ZnS Quantum dots with Tunable Crystal structure and Photoluminescent Properties. Nanomaterials 12. 10.3390/nano12172969 10.3390/nano12172969 PMC9457710 36080006

[49] Jasieniak J, Mulvaney P (2007) From Cd-rich to Se-rich - the manipulation of CdSe nanocrystal surface stoichiometry. J Am Chem Soc 129:2841–2848. 10.1021/JA066205A/SUPPL_FILE/JA066205ASI20061214_063807.PDF 17309253 10.1021/ja066205a

[50] Gao Y, Peng X (2015) Photogenerated excitons in plain core CdSe nanocrystals with unity radiative decay in single channel: the effects of surface and ligands. J Am Chem Soc 137:4230–4235. 10.1021/jacs.5b01314 25785631 10.1021/jacs.5b01314

[51] La Rosa M, Denisov SA, Jonusauskas G et al (2018) Designed Long-Lived Emission from CdSe Quantum Dots through Reversible Electronic Energy Transfer with a surface‐bound chromophore. Angew Chem 130:3158–3161. 10.1002/ange.201712403 10.1002/anie.201712403 PMC5873259 29383800

[52] Kempken B, Dzhagan V, Zahn DRT et al (2015) Synthesis, optical properties, and photochemical activity of zinc-indium-sulfide nanoplates. RSC Adv 5:89577–89585. 10.1039/c5ra20570k

[53] Du Fossé I, Ten Brinck S, Infante I, Houtepen AJ (2019) Role of Surface reduction in the formation of traps in n-Doped II-VI Semiconductor nanocrystals: how to charge without reducing the Surface. Chem Mater 31:4575–4583. 10.1021/acs.chemmater.9b01395 31274957 10.1021/acs.chemmater.9b01395 PMC6595709

[54] Zhu C, Li L, Fang F et al (2005) Functional InP nanocrystals as novel near-infrared fluorescent sensors for mercury ions. Chem Lett 34:898–899. 10.1246/cl.2005.898

[55] Boonmee C, Noipa T, Tuntulani T, Ngeontae W (2016) Cysteamine capped CdS quantum dots as a fluorescence sensor for the determination of copper ion exploiting fluorescence enhancement and long-wave spectral shifts. Spectrochim Acta Mol Biomol Spectrosc 169:161–168. 10.1016/J.SAA.2016.05.007 10.1016/j.saa.2016.05.007 27372512

[56] Ding Y, Shen SZ, Sun H et al (2014) Synthesis of l-glutathione-capped-ZnSe quantum dots for the sensitive and selective determination of copper ion in aqueous solutions. Sens Actuators B Chem 203:35–43. 10.1016/J.SNB.2014.06.054

[57] Ben Brahim N, Poggi M, Lambry JC et al (2018) Density of grafted chains in Thioglycerol-Capped CdS Quantum dots determines their Interaction with Aluminum(III) in water. Inorg Chem 57:4979–4988. 10.1021/ACS.INORGCHEM.7B03254/SUPPL_FILE/IC7B03254_SI_001.PDF 29648807 10.1021/acs.inorgchem.7b03254

