# Supplementary material for: Influence of Structural Properties of Oleic Acid-Capped CdSe/ZnS Quantum Dots in the Detection of Hg2+ Ions
Source: J Fluoresc. 2024 Jul 16;35(6):4385–97. doi: 10.1007/s10895-024-03828-0 (PMC12206198; doi:10.1007/s10895-024-03828-0)
Supplement: Supplementary file 1 — Supplementary Material 1 [file 10895_2024_3828_MOESM1_ESM.docx]

**SUPPORTING INFORMATION**

**Influence of structural properties of oleic acid-capped CdSe/ZnS quantum dots in the detection of Hg^2+^ ions**

Fredy Giovany Ortiz Calderon,^1,2^ Brayan Stiven Gómez Pineros,^1^ Nathan D. McClenaghan,^2^ Gilma Granados-Oliveros^1^*

^1^ Grupo de Síntesis Orgánica Sostenible, Departamento de Química, Facultad de Ciencias, Universidad Nacional de Colombia, Bogotá, D. C.; Colombia

^2^ Institut des Sciences Moléculaires, CNRS UMR 5255, University of Bordeaux, 33405 Talence, France

**1. Synthesis of oleic-acid CdSe/ZnS QD^1^**

CdSe core synthesis. 34.2 mg (0.25 mmol) of CdO, 0.6 mL (1.9 mmol) of oleic acid, and 10 mL of ODE were mixed into a 250 mL three-neck flask under N_2_ atmosphere. The mixture was heated at 225 °C under magnetic stirring for 90 min. In these conditions, CdO was dissolved. In another three-neck flask and under N_2_ atmosphere, Se/TOP was prepared by mixing 30.5 mg (0.38 mmol) of selenium, 5 mL of ODE, and 0.45 mL (0.90 mmol) of TOP solution and heated at 80 °C for 3 h. Then, 1.0 mL Se/TOP was quickly injected into CdO solution at 225 °C for CdSe nucleus growth for 48 s, and at that moment, the temperature was adjusted to 100 °C.

CdSe/ZnS synthesis. A Zn/S/TOP solution was prepared by mixing 31 μL (0.031 mmol) of Zn(C_2_H_5_)_2_ solution, 64 μL (0.28 mmol) of (TMS)_2_S, and 1 mL (2 mmol) of TOP under N_2_ atmosphere. This solution was added dropwise to CdSe solution under magnetic stirring, keeping N_2_ atmosphere, and heated to 80 °C for 4 h. This procedure was repeated twice more, using 140 μL (0.14 mmol) and 280 μL (0.28 mmol) of Zn(C_2_H_5_)_2_. At the same time, the amount of (TMS)_2_S was kept constant (64 μL, 0.28 mmol).

After the ZnS shell formation around CdSe core, purification was carried out by precipitation of QDs with 10 mL of cold chloroform. Separation of solid was done by centrifugation at 15000 rpm for 25 min. The resulting solid was redissolved in chloroform, heated at 80 °C, and precipitated by dropwise addition of acetonitrile. This purification process was repeated 3 times.

References

[1] Granados-Oliveros G, Pineros BSG, Calderon FGO (2022) CdSe/ZnS quantum dots capped with oleic acid and L-glutathione: Structural properties and application in detection of Hg^2+^. J Mol Struct 1254:132293. https://doi.org/10.1016/J.MOLSTRUC.2021.132293

**Figure S1.** TEM and HR-TEM (inset) micrographs and distribution of size (histograms) of CdSe core QDs


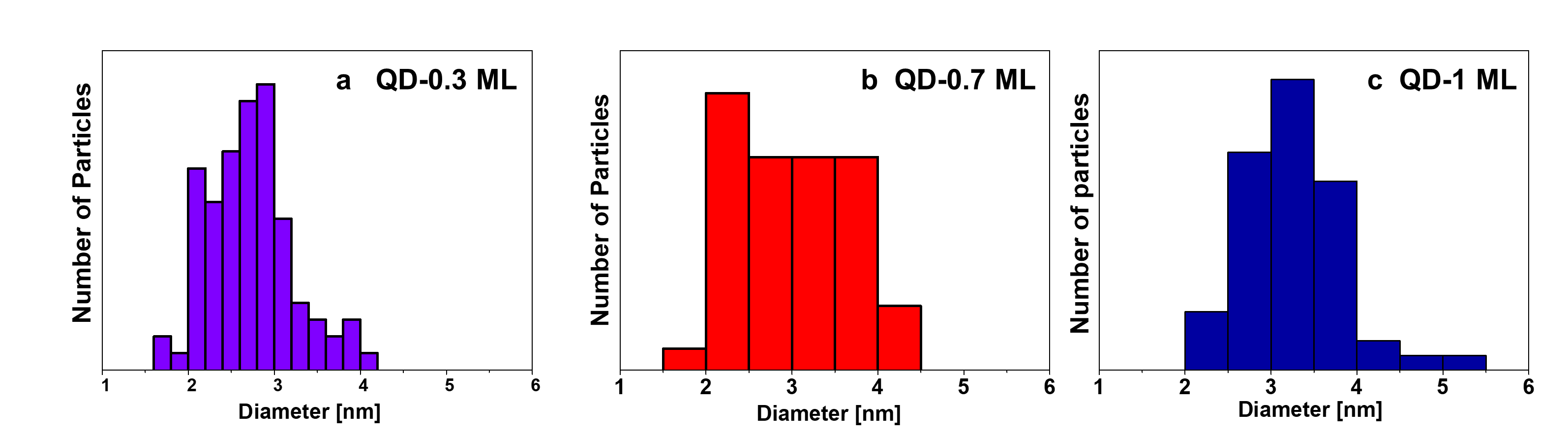


**Figure S2**. Distribution of size (histograms) of CdSe/ZnS QDs: (a) QD-0.3 ML, (b) QD-0.7 ML and (c) QD-1 ML

**Figure S3.** Typical XPS survey spectra of (A) CdSe and (B) CdSe/ZnS QDs
